# Simulation of Laser Heating of Aluminum and Model Validation via Two-Color Pyrometer and Shape Assessment

**DOI:** 10.3390/ma11091506

**Published:** 2018-08-22

**Authors:** Fabrizia Caiazzo, Vittorio Alfieri

**Affiliations:** Department of Industrial Engineering, University of Salerno, 84084 Fisciano, Italy; valfieri@unisa.it

**Keywords:** modeling, laser processing, simulation, pyrometer, aluminum

## Abstract

The modeling of laser-based processes is increasingly addressed in a competitive environment for two main reasons: Preventing a trial-and-error approach to set the optimum processing conditions and non-destructive real-time control. In this frame, a thermal model for laser heating in the form of non-penetrative bead-on-plate welds of aluminum alloy 2024 is proposed in this paper. A super-Gaussian profile is considered for the transverse optical intensity and a number of laws for temperature-dependent material properties have been included aiming to improve the reliability of the model. The output of the simulation in terms of both thermal evolution of the parent metal and geometry of the fusion zone is validated in comparison with the actual response: namely, a two-color pyrometer is used to infer the thermal history on the exposed surface around the scanning path, whereas the shape and size of the fusion zone are assessed in the transverse cross-section. With an average error of 3% and 4%, the model is capable of predicting the peak temperature and the depth of the fusion zone upon laser heating, respectively. The model is intended to offer a comprehensive description of phenomena in laser heating in preparation for a further model for repairing via additive manufacturing.

## 1. Introduction

Simulation tools are crucial in a competitive environment to prevent a trial-and-error approach to set the optimum processing conditions at a pre-design stage [1]. Moreover, proper modeling of an industrial process is the key to introduce closed-loop real-time monitoring where signals are managed for the purpose of control [2], to correct possible deviations of the main factors with respect to the intended, simulated response. Therefore, two issues must be addressed: the building of a reliable model structure and the arranging of effective equipment for real-time monitoring.

Regarding the former, the need for developing simulation tools to predict the transient temperature fields in laser-based processes has been widely presented in the literature [3]. Indeed, irrespective of the application and the technology, it has been shown that temperature directly affects the mechanical properties of the final component.

Some effort has been made in this field and a wide range of applications are reported in the literature, including but not limited to hardening [4], laser ablation [5], laser cutting [6], laser drilling [7], laser welding [8,9], and additive manufacturing of metal powder [10,11]. Irrespective of the application, the prediction of the temperature field is crucial for many purposes, including but not limited to non-destructive real-time evaluation of the process [2], minimization of residual stresses, and heat accumulation during additive manufacturing [11,12]. In general, advanced complex models are required to consider beam attenuation in the laser-induced plasma plume when higher irradiance is delivered (e.g., in cutting, drilling, and welding). Specific additional references to common assumptions for modeling will be given in the following relevant sections of the paper.

Once a proper simulation tool has been developed to relate the input parameters on the laser thermal cycles, signals must be extracted from the process and continuously compared to the intended output. Therefore, for the purpose of exploiting the transient temperature field in real-time monitoring and control, a cost-effective, fast, and reliable solution should aim to reduce the error in temperature measurements. A number of methods and detection sensors have been proposed in the literature: Thermocouples, photodiodes, and infrared cameras are the main methods that have been tested and compared [13]. Unfortunately, they are generally unsuitable for laser-based processes in an industrial environment, since fast measurements at precise locations approaching the laser path are required. Indeed, sharp temperature gradients as a consequence of fast heating and cooling rates are involved in laser processing; moreover, the acquisition may be significantly affected by laser radiation and plume dynamics, depending on the metal to be processed and the operating window [2].

Instead, fiber-optic pyrometers are a valuable method, being contactless and faster, with a response time in the order of milliseconds [14]. Additional advantages are offered by two-color pyrometers [15] dealing with the ratio of optical powers at two spectral bands to bypass the dependence of emissivity on the temperature. A wide theoretical background about detecting with two-color pyrometers is available in the literature [16].

A model for laser thermal heating in the form of non-penetrative bead-on-plate welds was built in COMSOL Multiphysics in this study, aiming to simulate the creation of the melting pool due to laser heating, in preparation for a further model where impinging metal would be fed for repair via additive manufacturing. Although some effort has been made in the literature to model the process of material deposition for the purpose of additive manufacturing [17,18], many simplifying assumptions are usually made in terms of material properties and boundary conditions, given that many complex phenomena are involved; in this frame, this paper specifically aimed to build a comprehensive model via a methodical approach contemplating several items. The validation of the process of simulating mere thermal heating (i.e., bead-on-plate welds) and the creation of a melting pool was required before moving to a more complex model; with this respect, the results are discussed in this paper. Namely, aluminum alloy (AA) 2024-T3 was chosen as a base metal, as it is widely used in the aerospace and automotive industries for high price-sensitive parts requiring maintenance via focused heat sources, such as laser beams. The reliability of the simulation was assessed in comparison with the experimental data, i.e., the thermal history around the scanning path and the geometrical response in the fusion zone upon cross-cutting. Namely, a fiber-optic two-color pyrometer was used to obtain temperature measurements.

## 2. Materials and Methods

### 2.1. Thermal Modeling

#### 2.1.1. Heat Equations

Heat generated by a laser beam above a metal surface is dissipated by means of conduction, convection, and radiation. The theoretical approach to modeling is provided in the literature [11]. Namely, the heat transport equation can be given as:

(1) $$\rho c\frac{\partial T}{\partial t}=\nabla \cdot \left(k\nabla T\right)+\alpha Q$$

where *ρ* is the density, *c* is the heat capacity, *T* is the temperature, *t* is the time, *k* is the thermal conductivity, *α* is the absorption coefficient, and *Q* is the laser heat generation. In addition, convection and radiation losses *q*_c_ and *q*_r_ are given as:

(2) $$q_{c}=h\left(T_{\infty }-T\right)$$

(3) $$q_{r}=\varepsilon \sigma \left(T_{\mathrm{room}}^{4}-T^{4}\right)$$

where *h* is the heat convection coefficient, *ε* is the emissivity, *σ* is the Stefan-Boltzmann constant, and *T*_∞_ and *T*_room_ are the gas medium and room temperature, respectively. In the following sections, losses will be provided in form of boundary conditions, depending on the domain of interest; both *T*_∞_ and *T*_room_ will be assumed as 22 °C.

#### 2.1.2. Heat Source

**Figure 1:**
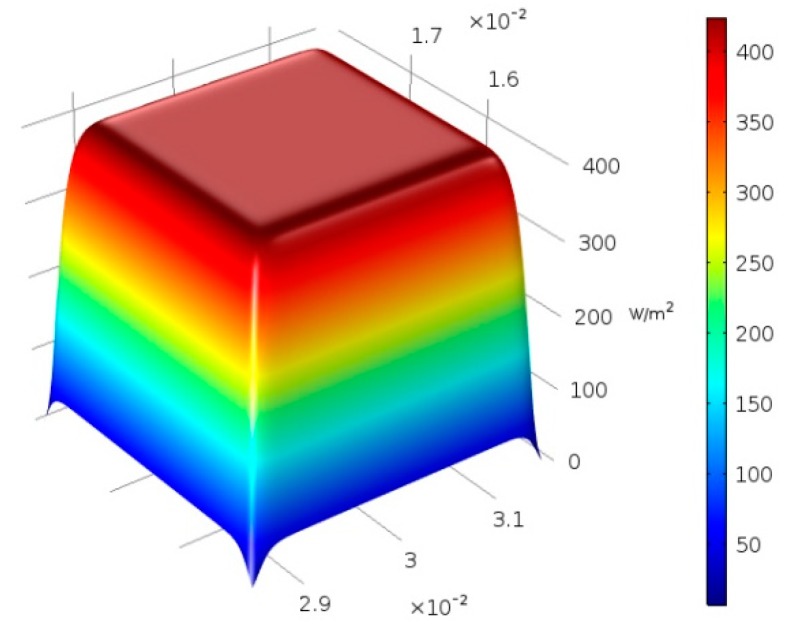
Distribution of transverse optical intensity: Super-Gaussian profile of order 20.

The first step to address is a proper description of the laser heat generation. The fundamental mode of a Gaussian beam [19] is generally preferred [11,20] and a Gaussian heat source is provided, accordingly. It is worth noting that although a lean description is gained, the assumption of a true Gaussian beam is not suitable in general, unless high-quality laser beams are considered. Other theoretical formulations are hence proposed in the literature, including a double-ellipsoid power density distribution [17] based on the original model suggested by Goldak [21], and a flat-top beam [19]. The latter is considered in this paper; namely, the heat generated by a super-Gaussian profile (i.e., a smoothed flat-top profile) of transverse optical intensity of order *n* can be given as:

(4) $$Q\left(r\right)=Q_{0}\exp\left[-2{\left(\frac{r}{w_{0}}\right)}^{n}\right]$$

where *Q*_0_ is the peak intensity, *w*_0_ is the beam radius over the incident surface, and *r* is the radial distance from the propagation axis. A conventional Gaussian profile results from a super-Gaussian one of order two; the higher the order, the steeper the edges of the profile. A super-Gaussian intensity profile of order 20 was implemented in the paper (Figure 1), based on actual data acquisition via beam profiler. Under this assumption and for *P* denoting the operating power, the peak intensity in Equation (4) approaches:

(5) $$Q_{0}=\frac{P}{\pi w_{0}^{2}}$$

Moreover, since the model is aimed to simulate an application of repairing via metal addition where a defocused beam must be used [22], the laser beam was defocused to a processing diameter of 3 mm.

With *x*_0_ and *y*_0_ being the coordinates of the starting point of the beam path, *s* the traveling speed of the laser beam along the *x*-direction, and *t* the time; a moving heat source was implemented in a Cartesian coordinate system, hence Equation (4) yielding:

(6) $$Q\left(x,y\right)=\frac{P}{\pi w_{0}^{2}}\exp\left\{-\frac{2\left[{\left(x-x_{0}\right)}^{20}+{\left(y-y_{0}\right)}^{20}\right]}{w_{0}^{20}}\right\}=\frac{P}{\pi w_{0}^{2}}\exp\left\{-\frac{2\left[{\left(s\;t-x_{0}\right)}^{20}+{\left(y-y_{0}\right)}^{20}\right]}{w_{0}^{20}}\right\}$$

#### 2.1.3. Material Properties

Including temperature-dependent material properties in the model is the key to a reliable prediction of the temperature field. For this purpose, based on the available literature on the characterization of pure aluminum and its alloys, a number of laws have been included in the background for the properties involved in conduction, convection, and radiation equations. AA 2024 of typical composition [23] for wrought products is used; solidus and liquidus temperature of 775 and 911 K, respectively, are given.

At first, a functional form for density (kg·m^−3^) is borrowed [24], depending on the aggregation status:

(7) $$\{\begin{array}{l} \rho_{solid}\left(T\right)=2813+0.03\times T-7.4\times 10^{-4}\times T^{2}+10^{-6}\times T^{3}-5.7\times 10^{-10}\times T^{4}\;T\leq 775\;K\\ \rho_{liquid}\left(T\right)=2725-0.32\times T\;T\geq 911\;K \end{array}$$

Within the solidification range between solidus and liquidus temperature, a general rule of mixtures (i.e., a two-phase model) is implemented:

(8) $$\rho =\theta_{solid}\rho_{solid}+\left(1-\theta_{solid}\right)\rho_{liquid}\;775<T<911\;K$$

with *θ_solid_* denoting the solid volume fraction. Regarding the heat capacity (J·kg^−1^·K^−1^), a similar approach is taken [25,26]:

(9) $$c_{solid}\left(T\right)=199+3.9\times T-7.4\times 10^{-3}\times T^{2}+5.2\times 10^{-6}\times T^{3}\;T\leq 775\;K$$

Constant extrapolation has been set to extend this law beyond the solidus temperature. An evolution of thermal conductivity (W·m^−1^·K^−1^) as a function of temperature in a solid state is available in the literature [26]. Based on this, a functional form has been extracted and implemented:

(10) $$k_{solid}\left(T\right)=137+2.9\times 10^{-4}\times T+1.3\times 10^{-6}\times T^{2}\;T\leq 775\;K$$

Linear extrapolation has been set to extend this law beyond the solidus temperature.

**Figure 2:**
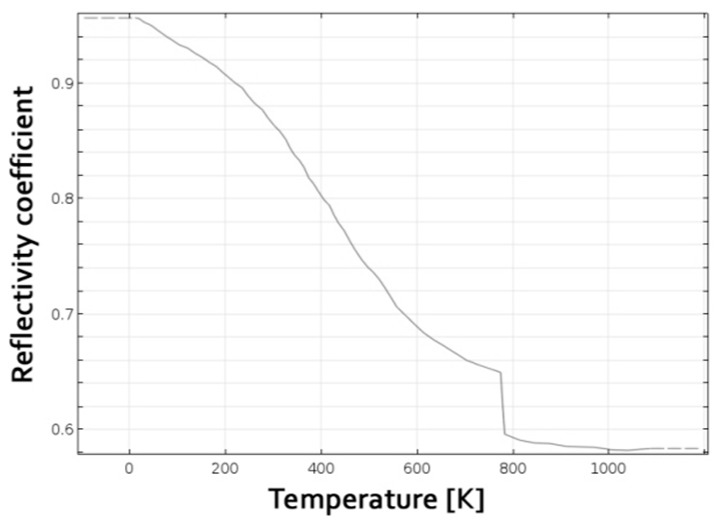
Aluminum reflectivity coefficient as a function of temperature.

For an opaque material, the absorption coefficient α is complementary to the reflection coefficient (1–α). Since reflection is one of the main factors affecting the coupling efficiency when processing metals [27], the trend of reflectivity vs. temperature is required: although constant average absorption has been proposed by some authors in the literature [11,18], the reflectivity of aluminum in solid state may decrease from 95 to as low as 60% [28]. Given this, a functional form for reflectivity, for a given operating laser wavelength of a doped YAG (Yttrium Aluminum Garnet) active gain, has been inferred (Figure 2), based on two main assumptions: a reduction of reflectivity and, hence, an increase of absorption, in turn, is reported for increasing temperature [28]; a sharp drop at a measure of 5% is noticed at phase transition [29] for pure aluminum.

In the assumption of natural convection, a constant heat convection coefficient *h* = 10 W·m^−2^·K^−1^ was fed to the model [17,20]. In the operating range of laser heating, even the dependence of emissivity on the temperature is mild: a constant value *ε* = 0.15 [30] was set. Eventually, generation of plasma, and, hence, beam attenuation, can be neglected since vaporization is prevented in this application [28].

#### 2.1.4. Virtual Specimen and Meshing

**Figure 3:**
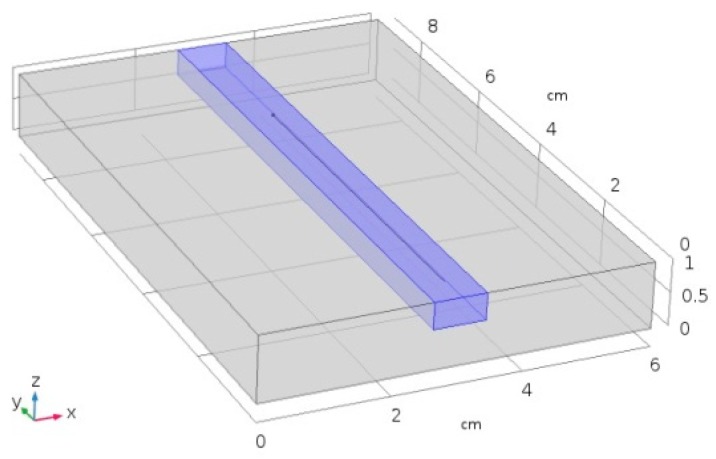
Domains of interest: central slot (blue domain, *D*_1_), and remaining (grey domain, *D*_2_).

**Figure 4:**
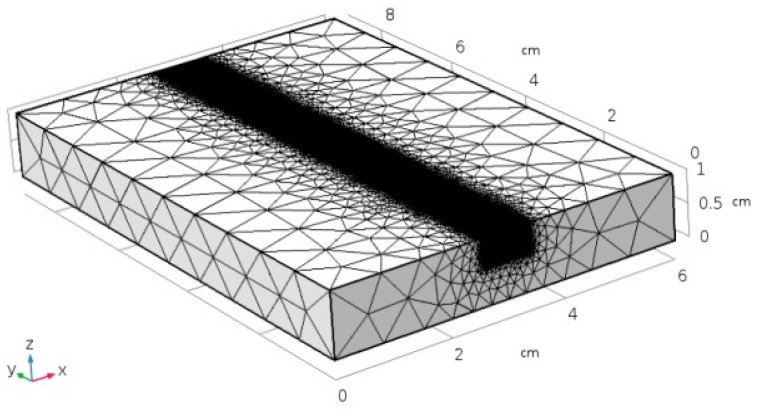
Triangle mesh of variable size on the virtual specimen.

**Figure 5:**
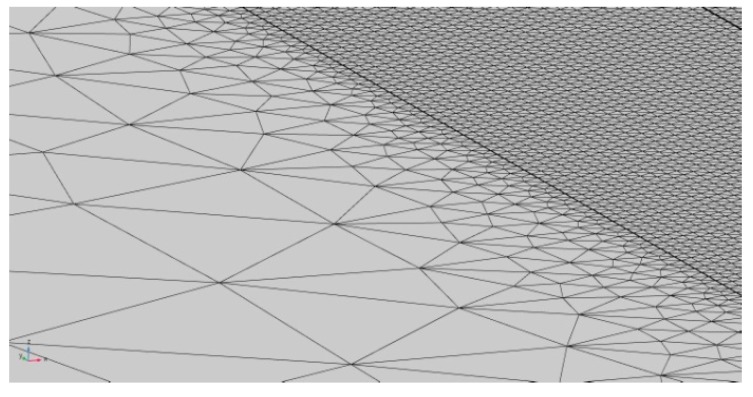
Mesh detail at the interface with the central slot.

Laser heating was modeled over a virtual specimen, 80 mm long, 60 mm wide, 10 mm thick, this being the size of the plate in the experimental procedure for validation. For the purpose of improving the consistency of the model, the specimen was divided into two domains of interest (Figure 3): a central, 10 mm wide, 0.4 mm deep slot for the laser path (domain 1, *D*_1_) and the remaining (domain 2, *D*_2_). Indeed, it is worth noting that the main issue in the modeling of a laser-based process is a reliable implementation of a processing diameter in the order of tenths of millimeters [19]. As a consequence of this, an ultrafine mesh must be set along the laser path, whereas a coarser mesh is allowed for the purpose of reducing the simulation time: a triangle mesh of variable size was applied, accordingly (Figure 4), the edges being 0.15 mm in size within *D*_1_ then ranging up to 10 mm across *D*_2_ (Figure 5).

Boundary and initial conditions are given for each domain. Regarding *D*_1_, laser heat generation *Q* is provided along the processing path; convection and radiation losses are experienced at the upper surface. Regarding *D*_2_, convection and radiation losses are experienced at each surface; a condition of thermal continuity is given with respect to *D*_1_ for each shared surface. The initial temperature of the domains is assumed as room temperature.

### 2.2. Experimental Procedure

Laser heating in the form of bead-on-plate tests was performed. The operating window to achieve effective penetration in the cross-section was borrowed from previous works on the same alloy [22], even aiming for non-penetrative bead-on-plate welds to prevent porosity [31]; traveling speed and power were considered, and the results of six testing conditions have been found (Table 1). For the purpose of an easier comparison of the responses, the length of the scanning path was conveniently set in order to result in 10 s heating, irrespective of the traveling speed.

**Figure 6:**
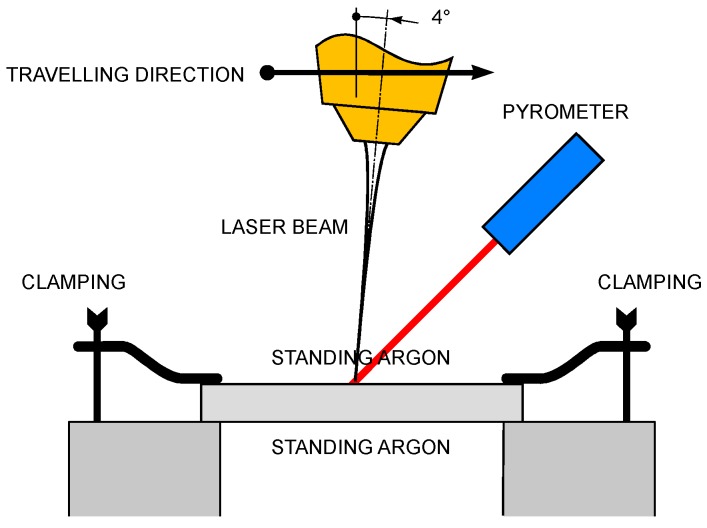
Scheme of the processing set-up; components are not to scale.

A thin-disc laser source was used (Table 2). Defocusing of the laser beam was set in order to get a processing diameter of 3 mm on the top surface. Moreover, a tilting angle of 4° was given to the laser head, in agreement with common practice for highly reflective metals, to prevent back-reflections from entering the optics train [27]; although reflectivity depends on the angle of incidence and the plane of polarization of the laser beam, the effect can be neglected at this angle size [28]. The scheme of the processing set-up was composed of a laser head, a clamping device, and a pyrometer (Figure 6).

Moreover, to prevent oxidation of the base metal, resulting in defects and additional heating as a consequence of energy release, argon for inert shielding was supplied to the working area; a steady shielding atmosphere resulted, giving grounds to the assumptions of natural convection in modeling. The plate was clamped at the edges, so that convection in standing argon was experienced and one may assume conduction is negligible to the purpose of simulating the thermal history at the laser path.

**Figure 7:**
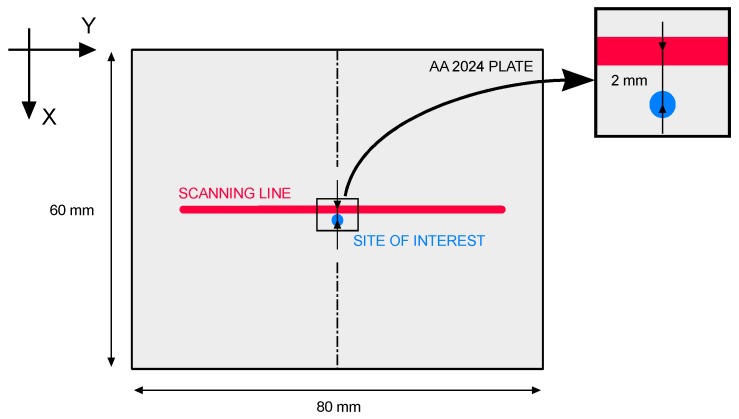
Location and detail of the site of interest (*x* = 32 mm, *y* = 40 mm, *z* = 10 mm) for thermal monitoring.

Temperature model validations were performed by means of a fiber-optic two-color pyrometer which was calibrated in a 290–610 °C (i.e., 563–883 K) temperature span to the specific purpose of monitoring AA 2024; as a consequence of this, underflow and overflow may result below and above the lower and higher span limits, respectively. For each given testing condition, the pyrometer was focused halfway in the processing path, 2 mm from the scanning line (Figure 7). The site of interest to focus the pyrometer is suggested by the need to acquire a response within the calibration range of the device; as a consequence, direct acquisition of the thermal history along the laser path is not feasible. A 0.1 ms time step was set for temperature acquisition, thus resulting in 10^5^ sample points overall, given a 10 s period of total heating. A post-acquisition smoothing algorithm with a 50-point moving-average was implemented in order to filter noise from the output.

**Figure 8:**
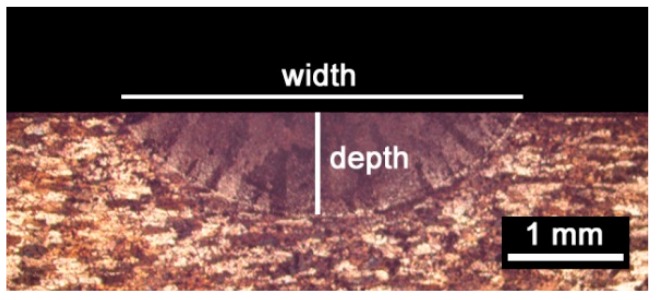
Width and depth of the fusion zone, macrograph resulting at speed of 6 mm/s, power of 2000 W.

Further data for model validation were obtained in the transverse cross-section (i.e., parallel to the *xz* plane). To this purpose, the specimens resulting from laser heating were cross-cut, mechanically ground, and polished to a mirror finish, and chemically etched with a solution consisting of 10% hydrofluoric acid, 15% nitric acid, and water at room temperature [23]. The actual size of the fusion zone was eventually measured via optical microscopy (Figure 8) and compared to the output of the simulation.

## 3. Results and Discussion

### 3.1. Local Thermal Cycle

**Figure 9:**
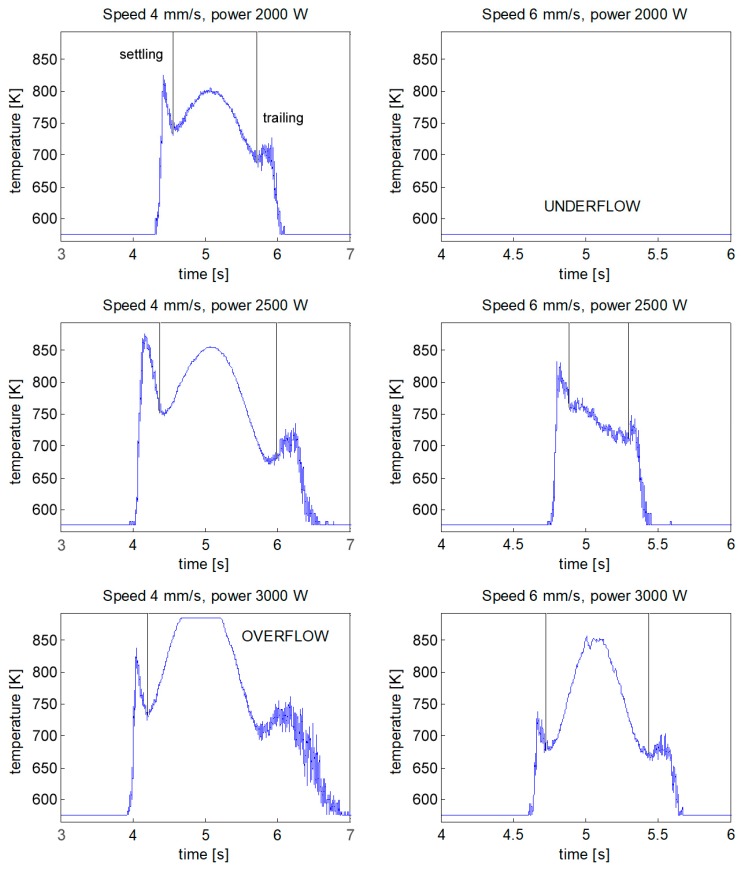
Thermal history of the site of interest as pyrometer output.

The thermal history at the site of interest during laser heating is a function of the processing parameters (Figure 9); depending on these, fusion may be experienced at the site of interest. The time-scale is started at laser switch-on: since the process duration is dependent on the traveling speed, the time to get the peak temperature at the site of interest (i.e., to cover half the distance) is 5 s.

A recurring shape was found for the temperature profile: namely, a settling period, resulting in a leading thermal spike, was required by the device when entering the window of calibration (i.e., the operating range of acquisition); a trailing noise was found at the end of the acquisition, due to the air and argon overheating over the site of interest, instead. The thermal evolution shifted below or above the calibration limits under extreme conditions of processing, thus resulting in underflow or overflow, respectively.

**Figure 10:**
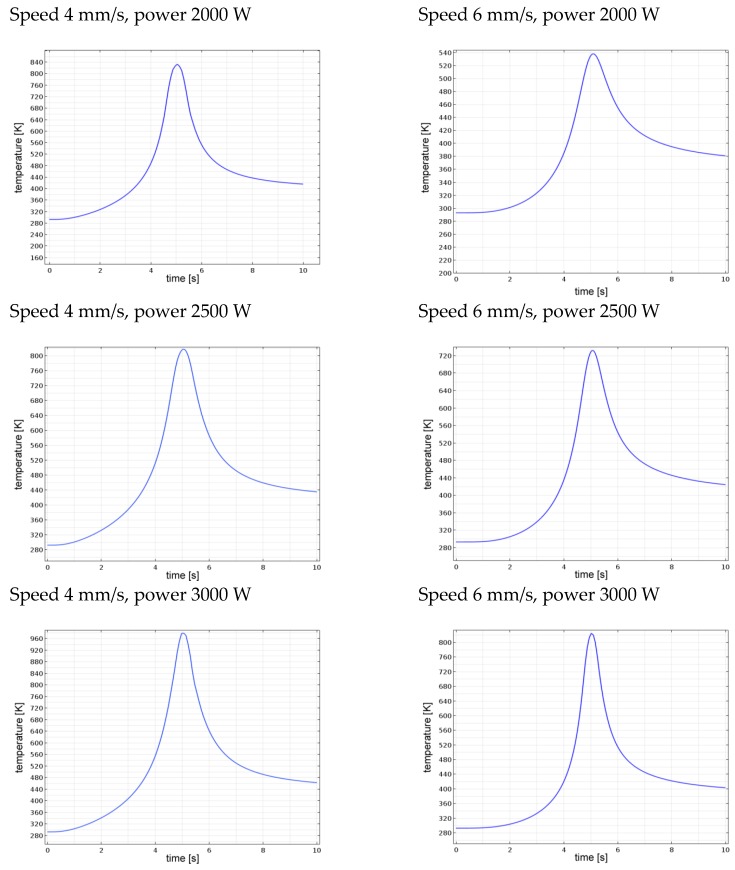
Simulated thermal history of the site of interest for each processing condition.

For each processing condition, the thermal evolution of the site of interest was simulated (Figure 10) and compared with the acquisition. The peak temperature (*Tp*) acquired by the pyrometer focus was extracted and compared with the predicted peak temperature in order to quantitatively validate the thermal model (Table 3); the percentage difference between acquisition and simulation was given. An agreement in a measure of 2.7%, absolute, on average, was found in terms of peak temperature; as regarding the conditions of underflow and overflow, the simulated thermal evolution was actually outside of the calibration window of the device.

### 3.2. Geometry of the Fusion Zone

**Figure 11:**
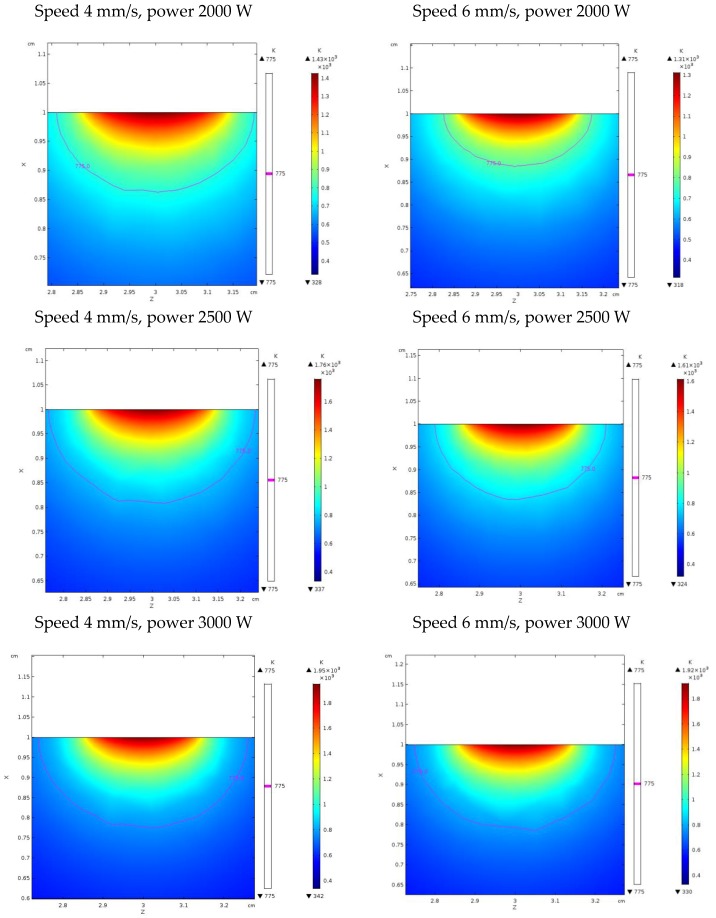
Contour lines to define the extent of the fusion zone for each processing condition.

Since the overall size of the fusion zone depends on the thermal history, further information to validate the model was gathered upon inspections in a transverse cross-section with respect to the traveling direction of the laser beam (Figure 8). An indirect measurement of the simulated depth of the fusion zone had to be conducted: namely, a transverse plane at half-length was considered with respect to the traveling direction, then thermal contour lines are drawn (Figure 11). As expected, any increase in the experienced peak temperature yielded a proportional increase in the extent of the fusion zone.

Based on the solidification range of the parent alloy, depth and width of the fusion zone were inferred. Indeed, since 775 K is the lower limit of the solidification range, fusion was experienced by any point above this temperature limit. For each given processing condition, the depth and width were compared to the corresponding actual geometry (Table 4). An agreement of 3.7% and 16.3%, absolute, on average, was found for depth and width, respectively. In order to improve the reliability of the model in predicting the width of the fusion zone, further investigation must be made and the dependence of the reflectivity on the starting roughness or oxide amount at the exposed surface should be considered.

## 4. Conclusions

A model to simulate laser heating was built and validated. The main elements were discussed and presented, aiming to offer a comprehensive description of the involved variables and phenomena. A super-Gaussian beam profile was implemented as an external thermal source; losses for radiation and convection were considered, whereas losses for plasma attenuation were neglected.

Under these assumptions, with an average error below 3%, the model is capable of predicting the peak temperature upon laser heating in a processing window ranging from 2 to 3 kW power and 4 to 6 mm/s speed, which is suitable to produce a melting pool where metal must be fed in additive manufacturing. With an average error below 4%, the model has been capable of predicting the depth of the fusion zone. Larger errors, of up to 16%, were found for the bead width instead; these will be addressed in future adjustments of the simulation; indeed, the dependence of the reflectivity on the starting roughness or oxide amount at the exposed surface should be considered. Furthermore, the model must be conveniently upgraded with powder or wire feeding to simulate layer-by-layer fabrication.

Interestingly, an industrial environment where a pyrometer output is used in real-time monitoring to match the intended thermal history, and, hence, the intended geometry, can be conceived. Nevertheless, proper actions both at software and hardware stages, must be taken to filter noise from the pyrometer output, depending on the system set-up, the metal to be processed, and the laser wavelength.

## Figures and Tables

**Table 1:** Processing conditions for laser heating.

Speed (mm/s)	Power (W)	Scanning length (mm)
4	2000	40
4	2500	40
4	3000	40
6	2000	60
6	2500	60
6	3000	60

**Table 2:** Main technical features of the laser source.

Parameter	Value
Maximum output power (kW)	4.0
Operating nominal wavelength (nm)	1030
Beam Parameter Product (mm × mrad)	8.0
M^2^ quality factor	24.3
Core diameter of the delivering fiber (µm)	300
Spot size of the laser beam on the surface (mm)	3.0

**Table 3:** Peak temperatures, actual vs. predicted.

Speed (mm/s)	Power (W)	*T_p_* (K)		
		Actual	Simulated	Difference
4	2000	803	815	1.5%
4	2500	848	828	−2.4%
4	3000	*overflow*	990	*n/a*
6	2000	*underflow*	543	*n/a*
6	2500	753	728	−3.3%
6	3000	851	821	−3.5%

**Table 4:** Depth and width of the fusion zone, actual vs. predicted.

Speed (mm/s)	Power (W)	Depth of Fusion (mm)			Width of Fusion (mm)		
		Actual	Simulated	Difference	Actual	Simulated	Difference
4	2000	1.55	1.42	−8.3%	5.17	4.22	−18.4%
4	2500	1.71	1.72	2.8%	5.45	4.53	−16.9%
4	3000	2.11	2.19	1.1%	6.81	5.55	−18.5%
6	2000	1.06	1.09	3.3%	4.06	3.57	−12.1%
6	2500	1.55	1.60	4.2%	5.13	4.38	−14.6%
6	3000	1.95	2.00	2.6%	6.21	5.10	−17.9%
